# Interaction of Hydrogen Sulfide and Estrogen on the Proliferation of Vascular Smooth Muscle Cells

**DOI:** 10.1371/journal.pone.0041614

**Published:** 2012-08-03

**Authors:** Hongzhu Li, Sarathi Mani, Wei Cao, Guangdong Yang, Christopher Lai, Lingyun Wu, Rui Wang

**Affiliations:** 1 Department of Biology, Lakehead University, Thunder Bay, Canada; 2 Department of Pathophysiology, Harbin Medical University, Harbin, China; 3 Department of Health Science, Lakehead University, Thunder Bay, Canada; 4 The School of Kinesiology, Lakehead University, Thunder Bay, Canada; 5 Northern Ontario School of Medicine, Lakehead University, Thunder Bay, Canada; University of Western Ontario, Canada

## Abstract

Hydrogen sulfide (H_2_S) can be endogenously generated from cystathionine gamma-lyase (CSE) in cardiovascular system, offering a cardiovascular protection. It is also known that the lower risk of cardiovascular diseases in female is partially attributed to the protective effect of estrogen. The current study explores the interaction of H_2_S and estrogen on smooth muscle cell (SMC) growth. In the present study, we found that the proliferation of cultured vascular SMCs isolated from wild-type mice (WT-SMCs) was inhibited, but that from CSE gene knockout mice (CSE-KO-SMCs) increased, by estrogen treatments. The expression of estrogen receptor α (ERα), but not ERβ, was significantly decreased in CSE-KO-SMCs compared with that in WT-SMCs. Exogenously applied H_2_S markedly increased ERα but not ERβ expression. In addition, the inhibition of ER activation and knockdown of ERα expression in WT-SMCs or the overexpression of ERα in CSE-KO-SMCs reversed the respective effects of estrogen on cell proliferation. The expression of cyclin D1 was reduced in WT-SMCs but increased in CSE-KO-SMCs after estrogen treatments, which was reversed by knockdown of ERα in WT-SMCs or overexpression of ERα in CSE-KO-SMCs, respectively. The overexpression of cyclin D1 in WT-SMCs or knockdown of cyclin D1 expression in CSE-KO-SMCs reversed the effects of estrogen on cell proliferation. These results suggest that H_2_S mediates estrogen-inhibited proliferation of SMCs via selective activation of ERα/cyclin D1 pathways.

## Introduction

Acquired proliferative phenotype of vascular smooth muscle cells (SMCs) is associated with development and progression of numerous vascular proliferative conditions, such as primary atherosclerosis and post-angioplasty restenosis [1]. Women generally experience initial manifestations of coronary artery disease 10 years later than men, suggesting that estrogen may offer a cardiovascular protection [2]. It is well known that estrogen inhibits the proliferation of SMCs [3]. Estrogen can bind to estrogen receptors (ERs), including ERα and ERβ, which are expressed in all vascular cell types and appear to mediate the inhibitive effects of estrogen on SMC proliferation [3], [4].

Estrogen activates many intracellular signaling responses [5]. Estrogen-stimulated mitogen-activated protein kinase (MAPK) cascade plays a key role in the cellular signal transduction pathway in response to vascular stimuli [6]. MAPK family in mammalian cells consists of three major members including ERK (an extracelluar signal-regulated kinase), JNK (c-Jun N-terminal kinase) and p38 MAPK [7]. Each of these MAPK plays a unique role in the regulation of gene expression and intracellular metabolism related to growth and development, apoptosis, and cellular responses to external stresses [7]. Cyclin D1 and its associated cyclin-dependent kinases (CDK4 and 6) are key regulatory proteins in controlling the reentry of quiescent cells from G0 into G1 [8]. Cyclin D1 was also reported to mediate the inhibitory effect of estrogen on cell proliferation [9].

Hydrogen sulfide (H_2_S) was traditionally viewed as a toxic gas detected in the contaminated environmental atmosphere [10]. Over the last decade, physiological importance of endogenously produced H_2_S has been realized [11]. Two key enzymes in the transsulfuration pathway, cystathionine beta-synthase (CBS) and cystathioine gamma-lyase (CSE), produce H_2_S, pyruvate and ammonium using homocysteine and/or L-cysteine as substrates [12], [13]. In some tissues, both CBS and CSE function to catalyze H_2_S production. CSE is the major H_2_S-producing enzyme in vascular tissues [14]. As an important gasotransmitter in the cardiovascular system, H_2_S has important physiological functions, such as anti-atherosclerosis, anti-inflammatory, vasodilatation, protection of ischemia injury, and antioxidant effects [12], [14]–[16].

Although both estrogen and H_2_S can inhibit SMC proliferation [3], [4], [7], [8], [13], the interaction of H_2_S and estrogen on SMC growth has been unknown. In the present study, we compared the effects of estrogen on the proliferation of SMCs from CSE knockout mice (CSE-KO-SMCs) and those from wild-type mice (WT-SMCs). The underlying cellular signaling mechanisms, including the activation of ERα, cyclin D1 and MAPK pathways, were further explored.

## Materials and Methods

### Materials

Estrogen (17β-estradiol) was from Sigma (St. Louis, MO). The anti-CSE antibody was purchased from Novus Biologicals (Littleton, CO). The anti-MAPK antibodies and different MAPK inhibitors were obtained from New England Biolabs (Camarillo, CA). Anti-cyclin D1 antibody was from Lab Vision Corporation (Fremont, CA). Anti-ERα, ERβ antibodies and siRNA for ERα as well as control siRNA were obtained from Santa Cruz Biotechnology (Santa Cruz, CA). Cyclin D1 siRNA and control siRNA were purchased from Ambion (Austin, TX). The plasmids pCMV-Cyclin D1 and pEGFP-C1-ERα were from Addgene (Cambridge, MA). Horseradish peroxidase-conjugated goat anti-rabbit IgG antibody and goat anti-mouse IgG antibody were from Sigma. All other chemicals were from Sigma or New England Biolabs.

**Figure 1:**
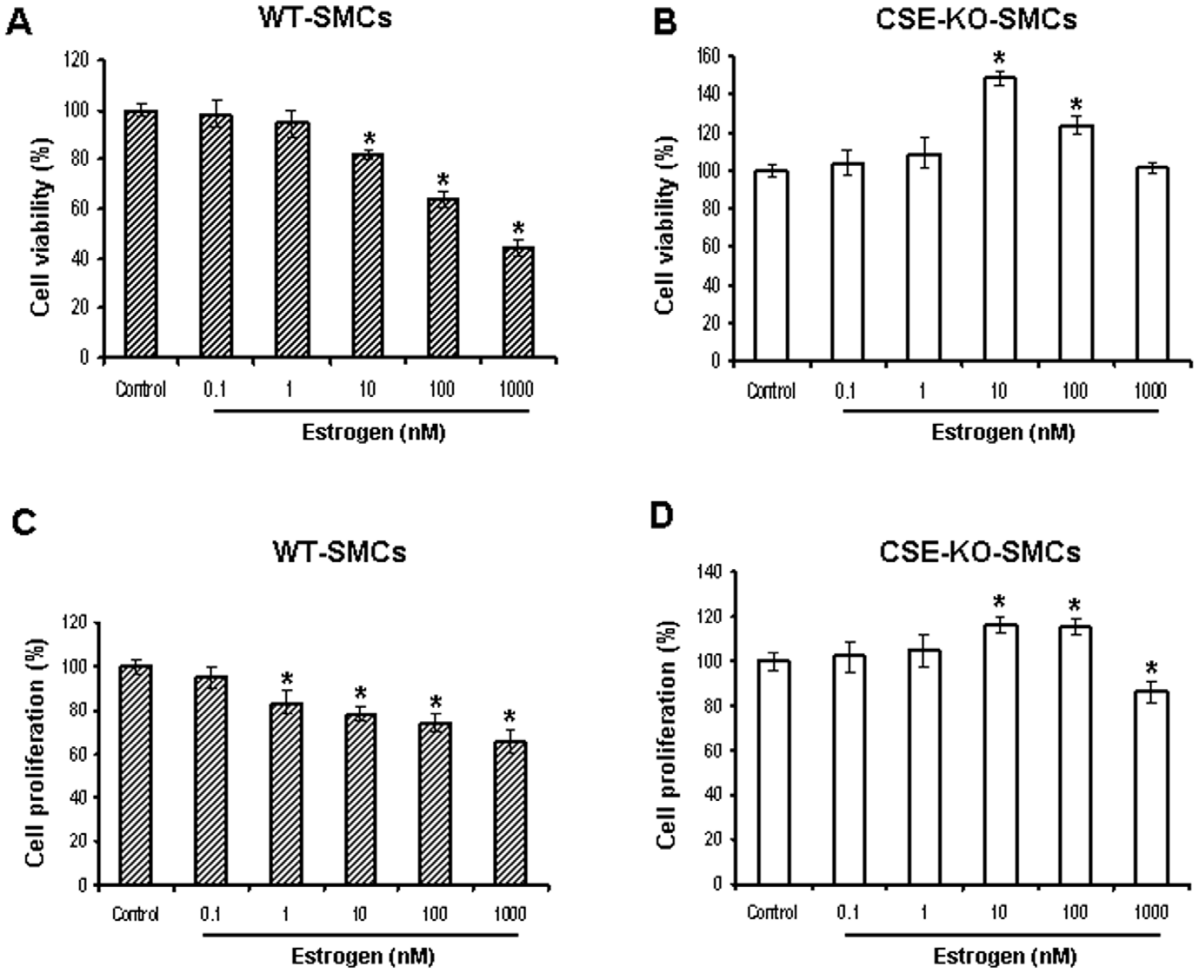
Inhibition of WT-SMC growth but promotion of CSE-KO-SMC growth by estrogen. WT-SMCs and CSE-KO-SMCs were treated with estrogen at the indicated concentrations for 72 h, and then cell viability and cell proliferation were measured. Cell viability of WT-SMCs (***A***) and CSE-KO-SMCs (***B***) were measured by MTT assay and cell proliferation of WT-SMCs (***C***) and CSE-KO-SMCs (***D***) were analyzed by BrdU incorporation assay. The control cells without estrogen treatment were considered as 100% viable. Data were from five independent experiments. * p<0.05 *vs*. control group.

### Isolation and Culture of SMCs

Twelve-week-old male CSE-KO offspring and age matched male WT littermates on C57BL/6J×129SvEv background were used to isolate primary SMCs from mesenteric arteries [17]. All animals were maintained on standard rodent chow and had free access to food and water. All animal experiments were conducted in compliance with the Guide for the Care and Use of Laboratory Animals published by the US National Institutes of Health (NIH Publication No. 85–23, revised 1996) and approved by the Animal Care Committees of Lakehead University, Canada. In brief, 12-week male WT and CSE-KO mice were generally anaesthetized by intraperitoneal injection with a mixture of 10 mg/kg xylazine (Bimeda-MTC Animal Health, CA) and 100 mg/kg ketamine hydrochloride (BIONICHE Animal Health, CA). When mice have lost muscle tone and blink reflexes, pupils become fixed and respiration is regular, the operation was carried out. Mice were killed by decapitation. Small mesenteric arteries below the second branch of the main mesenteric artery were dissected out and kept in ice-cold physiological salt solution (PSS) containing (in mM) NaCl 137, KCl 5.6, NaH_2_PO_4_ 0.4, Na_2_HPO_4_ 0.4, NaHCO_3_ 4.2, MgCl_2_ 1, CaCl_2_ 2.6, Hepes 10, and glucose 5 with pH adjusted to 7.4 with NaOH. The small arteries were cut into 5 mm-long pieces, and incubated at 37°C in low-Ca^2+^ PSS (0.1 mM CaCl_2_) containing 1 mg/ml albumin, 0.5 mg/ml papain, and 1 mg/ml dithioerythritol for 30 min, and for another 20 min in the Ca^2+^-free PSS containing 1 mg/ml albumin, 0.8 mg/ml collagenase, and 0.8 mg/ml hyaluronidase. Single cells were released by gentle trituration through a Pasteur pipette, stored in the nominally Ca^2+^-free PSS at 4°C, and used the same day. These primary SMCs were grown in phenol red-free Dulbecco’s modified Eagle’s medium (DMEM) supplemented with 10% fetal bovine serum (FBS), 100 U/ml penicillin, and 100 mg/ml streptomycin. The experiments were performed when the cells reached 70–80% confluence between passages 6 and 10. In all studies, cells were first incubated in the serum-free medium for 24 h and then 1% serum added together with different treatments, the media were changed every 2 days.

**Figure 2:**
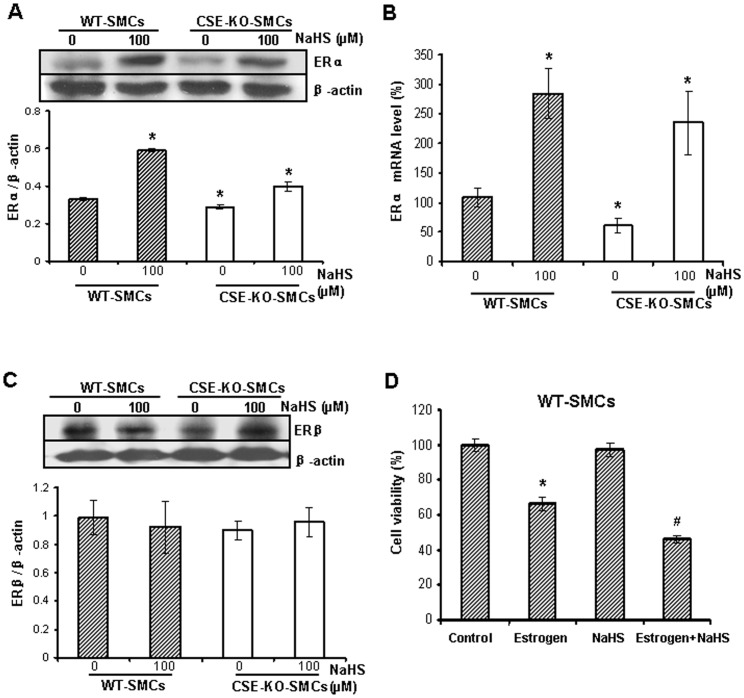
The effect of H_2_S on ERα and ERβ expression. *A* , NaHS up-regulated the protein expressions of ERα in both WT-SMCs and CSE-KO-SMCs. The cells were treated with NaHS at 100 µM for 24 h. * p<0.05 *vs*. WT-SMCs. ***B***, NaHS up-regulated the expression of ERα mRNA in both WT-SMCs and CSE-KO-SMCs. The cells were treated with NaHS at 100 µM for 24 h. * p<0.05* vs*. WT-SMCs. ***C***, NaHS had no effect on the protein expressions of ERβ in both WT-SMCs and CSE-KO-SMCs. ***D***, NaHS potentiated the inhibitory effect of estrogen on the growth of WT-SMCs. After WT-SMCs were treated with 100 nM estrogen for 72 h in the presence or absence of 100 µM NaHS, cell viability was measured. * p<0.05 *vs*. control group; ^#^ p<0.05 *vs*. estrogen group. All data were from three independent experiments.

### Cell Viability Assay

Cell viability was determined by the 3-(4,5-dimethylthiazol-2-yl)-2,5-diphenyltetrazolium bromide (MTT) assay as described previously [18]. In brief, equal numbers of cells were seeded into each well of 96-well plates for 24 h. After different treatments, MTT (final concentration, 5 mg/ml) was added to each well, and then the cells were incubated for 4 h at 37°C. The cell culture medium was removed, and dimethyl sulfoxide (200 µl/well) was added. The absorbance of formazan products at 570 nm was measured in a FLUOstar OPTIMA microplate spectrophotometer (BMG LABTEch, Offenburg, Germany). The cells incubated with control medium were considered 100% viable.

**Figure 3:**
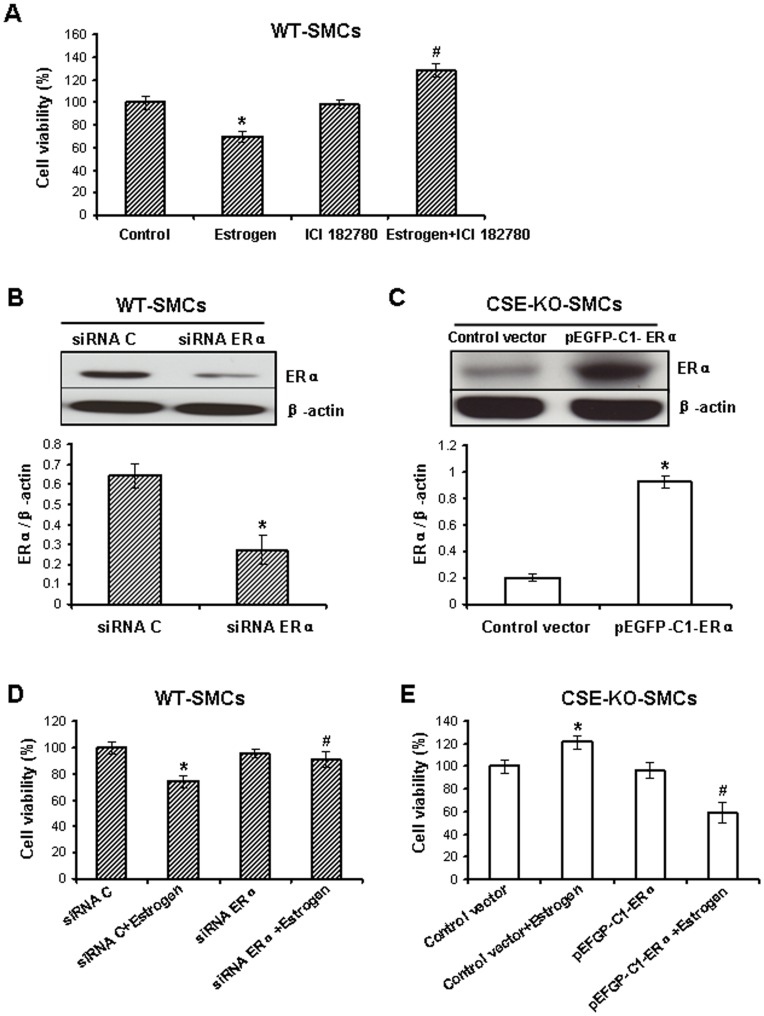
The role of ERα in **the actions of estrogen on the growth of WT-SMCs and CSE-KO-SMCs.** ***A*** **,** Blockage of ERs activity reversed estrogen-inhibited cell viability in WT-SMCs. The cells were treated with 10 µM ICI 182780 for 30 min following another 72 h treatment with 100 nM estrogen. * p<0.05 *vs*. control group; ^#^ p<0.05 *vs*. estrogen group. ***B***, Knockdown of ERα by ERα-specific siRNA (siRNA ERα) in WT-SMCs. The cells were transfected with 50 nM siRNA ERα or negative control siRNA (siRNA C) for 48 h. * p<0.05. ***C***, Overexpression of ERα in CSE-KO-SMCs. The cells were transfected with the plasmid pEGFP-C1-ERα or control plasmid pEGFP-C1 for 24 h. * p<0.05. ***D***, Knockdown of ERα reversed estrogen-decreased cell viability in WT-SMCs. The cells were transfected with siRNA ERα or siRNA C at 50 nM for 12 h following another 36 h treatment with estrogen (100 nM). * p<0.05 *vs*. siRNA C group; ^#^ p<0.05 *vs*. siRNA C+estrogen group. ***e***, Overexpression of ERα reversed estrogen-increased cell viability in CSE-KO-SMCs. The cells were transfected with pEGFP-C1-ERα plasmid or control plasmid for 24 h following another 48 h treatment with estrogen (100 nM). * p<0.05 *vs*. control vector group; ^#^ p<0.05 *vs*. control vector+estrogen group. All data were from four independent experiments.

### Cell Proliferation Assay

Cell proliferation was assessed by bromodeoxyuridine (BrdU) incorporation as described previously [8]. SMCs were plated at equal numbers of cells (2.0×10^3^ per well) in a 96-well plate for 24 h in the presence of 10% FBS. BrdU label was added into the culture media for another 24 h. Incorporated BrdU was detected following the procedures provided by the manufacturer (Calbiochem, Gibbstown, NJ, USA). For the experiments with estrogen treatments the cells were first seeded in a 96-well plate for 24 h in media containing 10% FBS, and then changed to a serum-free medium for another 24 h. Afterwards, 1% FBS was added back together with estrogen (0.1–1000 nM) for 48 h following BrdU label for additional 24 h.

### Measurement of H_2_S Production

H_2_S production rate was measured as described previously [19]. In brief, after different treatments, the cells were collected and homogenized in 50 mM ice-cold potassium phosphate buffer (pH 6.8). The flasks containing the reaction mixture (100 mM potassium phosphate buffer, 10 mM l-cysteine, 2 mM pyridoxal 5-phosphate, and 10% cell homogenates) and center wells containing 0.5 ml 1% zinc acetate and a piece of filter paper (2×2.5 cm) were flushed with N_2_ gas and incubated at 37°C for 90 min. The reaction was stopped by adding 0.5 ml of 50% trichloroacetic acid, and the flasks were incubated at 37°C for another 60 min. The contents of the center wells were transferred to test tubes, each containing 3.5 ml of water. Then 0.5 ml of 20 mM N, N-dimethyl-p-phenylenediamine sulfate in 7.2 M HCl and 0.5 ml 30 mM FeCl_3_ in 1.2 M HCl was added. The absorbance of the resulting solution at 670 nm was measured 20 min later with a FLUOstar OPTIMA microplate spectrophotometer.

**Figure 4:**
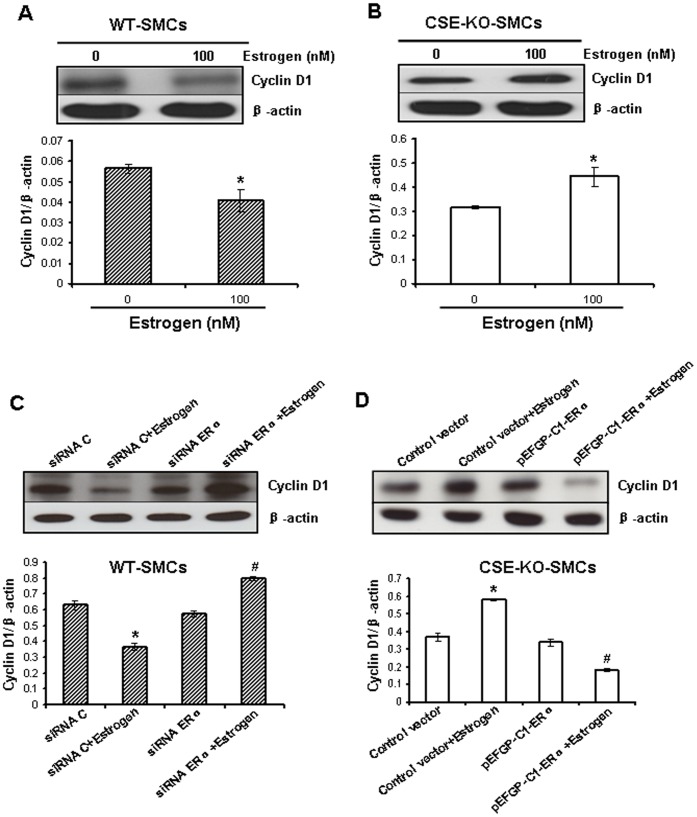
Estrogen altered the expression of cyclin D1. Estrogen reduced the expression of cyclin D1 in WT-SMCs (***A***) but up-regulated it in CSE-KO-SMCs (***B***). The cells were treated with 100 nM estrogen for 72 h. * p<0.05. ***C***, Knockdown of ERα reversed estrogen-decreased expression of cyclin D1 in WT-SMCs. After transfection with siRNA ERα or siRNA C at 50 nM for 12 h, the cells were incubated with 100 nM estrogen for another 36 h. * p<0.05 *vs*. siRNA C group; ^#^ p<0.05 *vs*. siRNA C or siRNA C+estrogen group. ***D,*** Overexpression of ERα reversed estrogen-increased expression of cyclin D1 in CSE-KO-SMCs. After transfection with pEGFP-C1-ERα plasmid or control plasmid for 24 h, the cells were incubated with 100 nM estrogen for another 48 h. * p<0.05 *vs*. control vector group; ^#^ p<0.05 *vs*. control vector or control vector+estrogen group. All data were from three independent experiments.

### Short Interfering RNA (siRNA) and Plasmid Transfection

Transfection of SMCs by siRNA or plasmids (pCMV-Cyclin D1 and pEGFP-C1-ERα as well as corresponding control plasmid) was achieved by using the Lipofectamine™ 2000 transfection agent from Invitrogen (Burlington, ON). In brief, SMCs were seeded at equal number of cells (2.0×10^5^ per plate) in 60 mm^2^ plates with the medium containing 10% FBS. The cells were plated to form 60–70% confluent monolayers for siRNA transfection, and 80–90% confluence for plasmid transfection. siRNA or plasmid and the transfection reagent complex were added to the serum-free medium for 4 h, and the transfection continued for another 48 h (for siRNA transfection) or 24 h (for plasmid transfection) in serum-containing regular medium. After that, the cells were collected for detection of protein expressions with western blotting analysis.

### Western Blotting Analysis

Cells were harvested and lysed. Equal amounts of proteins were boiled and separated with SDS-PAGE and electrophoretically transferred to a nitrocellulose membrane, as described previously [20]. In each lane of a 10% sodium dodecyl sulfate-polyacrylamide gel electrophoresis, equal amounts of proteins were applied, electrophoresed and transferred to a polyvinylidene fluoride membrane. Membranes were blocked with Tris-buffered saline containing 5% non-fat milk at room temperature for 1 h, then incubated overnight at 4°C with primary antibody. The primary antibody dilutions were 1∶1,000 for CSE, ERα, ERβ and Cyclin D1, phosphorylated or total ERK, p38 MAPK, or JNK, and 1∶10,000 for β-actin. The membrane was then washed three times with 1×Tris-buffer saline-Tween 20 (TBST) buffer and incubated in TBST solution with horseradish peroxidase-conjugated secondary antibody (diluted 1∶5,000) for 1 h at room temperature on a shaker. Finally, the membrane was washed with TBST solution for 3 times. The immunoreactions were visualized with ECL and exposed to x-ray film (Kodak Scientific Imaging film, Kodak, Rochester, NY).

**Figure 5:**
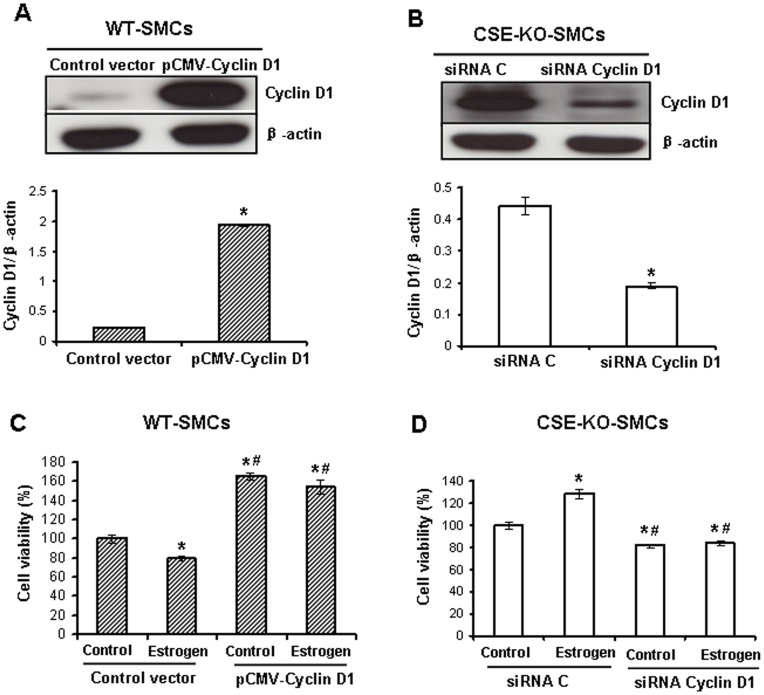
Cyclin D1 mediates estrogen-regulated cell proliferation in WT-SMCs and CSE-KO-SMCs. ***A***, Overexpression of cyclin D1 in WT-SMCs. The cells were transfected with the vector pCMV-Cyclin D1 or control vector pShuttle-CMV for 24 h. * p<0.05. ***B***, Knockdown of cyclin D1 in CSE-KO-SMCs. The cells were transfected with cyclin D1-specific siRNA (siRNA cyclin D1) or control siRNA (siRNA C) at 50 nM for 48 h. * p<0.05. ***C***, Overexpression of cyclin D1 reversed estrogen-inhibited cell viability in WT-SMCs. The cells were transfected with pCMV-Cyclin D1 or control vector for 24 h following another 48 h treatment with 100 nM estrogen. * p<0.05 *vs*. control vector+control group,^ #^ p<0.05 *vs*. control vector+estrogen group. ***D***, Knockdown of cyclin D1 reversed estrogen-induced cell viability in CSE-KO-SMCs. The cells were transfected with siRNA cyclin D1 or siRNA C at 50 nM for 12 h following another 36 h treatment with 100 nM estrogen. * p<0.05 *vs*. siRNA C control group, ^#^ p<0.05 *vs*. siRNA C+estrogen group. All experiments were repeated for four times.

### Real-time PCR Analysis

Total RNA was isolated using an RNeasy Mini Kit (Qiagen, Germantown, MD) and converted to cDNA with an iScriptTM cDNA Synthesis Kit (Bio-Rad, Hercules, CA). Real-time PCR was performed in an iCycler iQ5 apparatus (Bio-Rad) associated with the iCycler optical system software (version 3.1) using SYBR Green PCR Master Mix, as described previously [8]. The primers of ERα were 5′-ATTCTGACAATCGACGCCAG-3′ (forward) and 5′-GTGCATTGGTTTGTAGCTGG-3′ (reverse). The primers for β-actin were 5′-ATGGTGGGAATGGGTCAGAA-3′ (forward) and 5′-CTTTTCACGGTTGGCCTTAG-3′ (reverse). The PCR conditions were as follows: denaturation at 95°C for 60 s, followed by 40 cycles of denaturation at 95°C for 15 s, annealing at 55°C for 15s, and extension at 72°C for 45 s. Relative mRNA quantification was calculated by using the arithmetic formula “2-ΔΔCT”, where ΔCT is the difference between the threshold cycle of a given target cDNA and an endogenous reference β-actin cDNA.

### Statistical Analysis

All data were expressed as the mean ± SE and represented at least three independent experiments. Statistical comparisons were made using student’s *t*-test or one-way ANOVA followed by a post hoc analysis (Tukey test) where applicable. Significance level was set at p<0.05.

**Figure 6:**
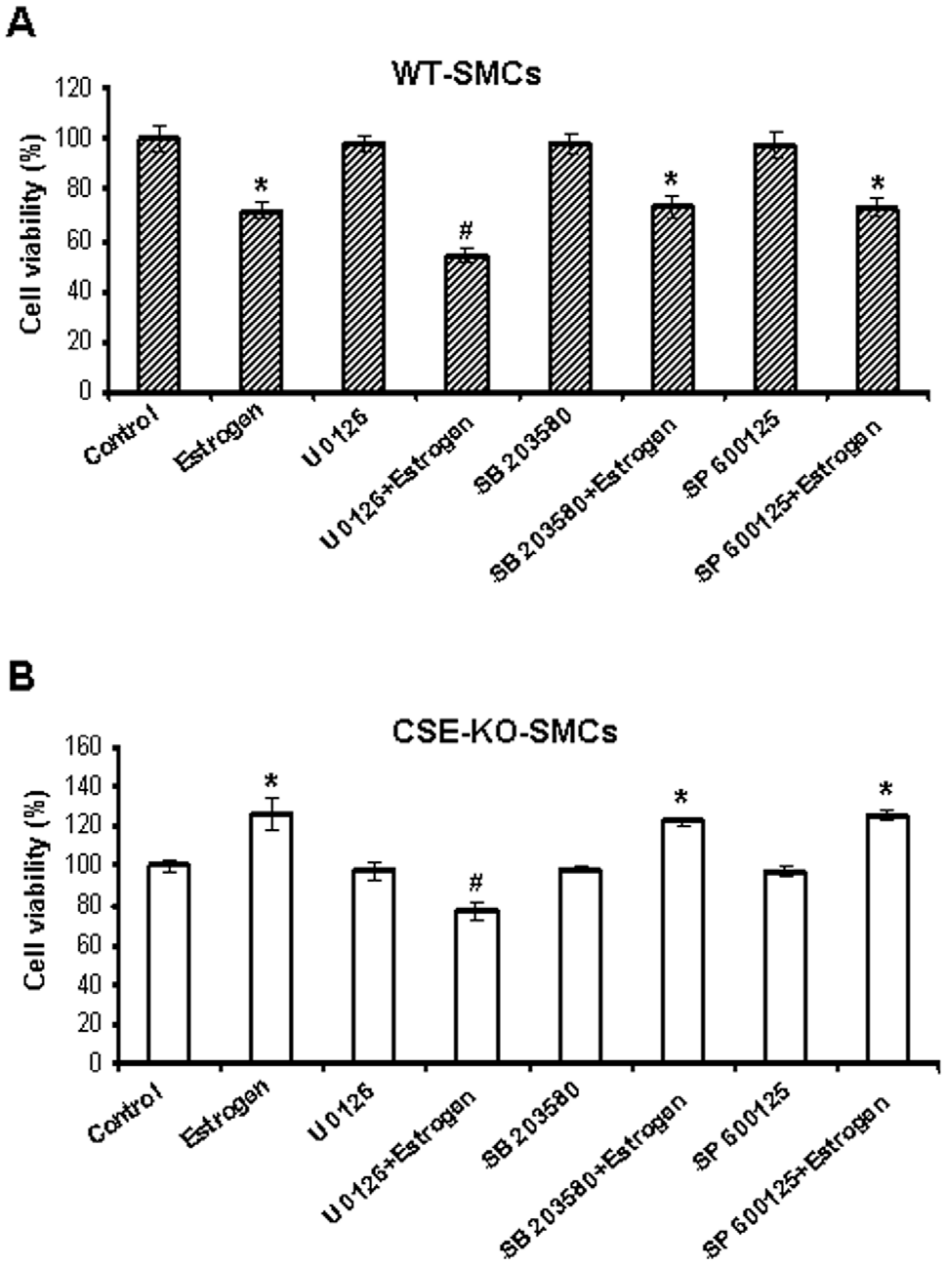
MAPK were not involved in estrogen-altered cell growth in WT-SMCs and CSE-KO-SMCs. WT-SMCs (***A***) and CSE-KO-SMCs (***B***) were pretreated with 10 µM U0126 (a MEK/ERK inhibitor), or 10 µM SB 203580 (a p38 MAPK inhibitor), or 10 µM SP 600125 (a JNK inhibitor) for 30 min followed by 100 nM estrogen for additional 72 h. The cell viability was evaluated by MTT assay. The data were from four independent experiments. * p<0.05 *vs*. control group; ^#^ p<0.05 *vs*. estrogen group.

## Results

### The Opposite Effects of Estrogen on the Growth of WT-SMCs and CSE-KO-SMCs

Estrogen at 10 to 1000 nM significantly decreased cell viability of WT-SMCs in a dose-dependent manner. However, at 10 to 100 nM estrogen significantly increased cell viability of CSE-KO-SMCs (Figure 1A and 1B). Cell viability data was further confirmed by cell proliferation analysis (Figure 1C and 1D). At the concentrations higher than 100 nM, the stimulatory effect of estrogen on proliferation of CSE-KO-SMCs could not be detected (Figure 1D).

### Effect of H_2_S on ERα Activation

The physiological functions of estrogen are mostly mediated by estrogen receptors [3], [4]. Our results showed that both mRNA and protein expressions of ERα were significant higher in WT-SMCs than that in CSE-KO-SMCs, and treatment with 100 µM NaHS (a H_2_S donor) for 24 h up-regulated ERα mRNA and protein expressions in both WT-SMCs and CSE-KO-SMCs (p<0.05) (Figure 2A and 2B). We further found that ERβ protein expression was not changed between WT-SMCs and CSE-KO-SMCs, and NaHS treatment had no effect on the expression of ERβ in both cells (p>0.05) (Figure 2C). In addition, NaHS at 100 µM potentiated the inhibitory effect of estrogen on cell viability of WT-SMCs (p<0.05) (Figure 2D). On the other hand, neither CSE expression nor H_2_S production rate was changed by estrogen treatments of WT-SMCs (p>0.05) (Figure S1A and S1B).

### The Role of ERα in Estrogen-altered Cell Growth of WT-SMCs and CSE-KO-SMCs

To explore whether estrogen receptor mediates the differential effects of estrogen on the growth of WT-SMCs and CSE-KO-SMCs, pretreatment of WT-SMCs with ICI 182780 (an ERs inhibitor) significantly reversed estrogen-inhibited cell viability (p<0.05), and ICI 182780 alone had no effect on cell viability (Figure 3A). Next we manipulated endogenous expression levels of ERα in both WT-SMCs and CSE-KO-SMCs. Compared with siRNA control group, siRNA ERα at 50 nM significantly inhibited ERα protein expression in WT-SMCs (p<0.05) (Figure 3B). The transfection of pEGFP-C1-ERα significantly overexpressed ERα protein in CSE-KO-SMCs when compared with pEGFP control vector (p<0.05) (Figure 3C). We further found that knockdown of ERα in WT-SMCs attenuated estrogen-inhibited cell viability in WT-SMCs, but overexpression of ERα in CSE-KO-SMCs reversed estrogen-enhanced cell viability (p<0.05) (Figure 3D and 3E).

### Estrogen-induced Down-regulation of Cyclin D1 in WT-SMCs but up-regulation in CSE-KO-SMCs

Cyclin D1 plays a critical role in the transition and progress of the cell cycle [8], [13]. Incubation of WT-SMCs with 100 nM estrogen for 72 h significantly decreased cyclin D1 expression (p<0.05) (Figure 4A). Quite differently, estrogen significantly increased the expression of cyclin D1 in CSE-KO-SMCs (Figure 4B). Knockdown of ERα in WT-SMCs or overexpression of ERα in CSE-KO-SMCs markedly reversed estrogen-altered cyclin D1 expression in respective cells (p<0.05) (Figure 4C and 4D). Furthermore, overexpression of cyclin D1 in WT-SMCs (Figure 5A) or knockdown of cyclin D1 in CSE-KO-SMCs (Figure 5B) reversed the effect of estrogen on cell viability (Figure 5C and 5D).

### Estrogen Activated MAPK in WT-SMCs and CSE-KO-SMCs

To determine whether estrogen differentially activates MAPK signaling pathways between WT-SMCs and CSE-KO-SMCs, we treated both cells with 100 nM estrogen for different durations. Estrogen treatment resulted in strong activation of ERK, p38 MAPK and JNK in both WT-SMCs and CSE-KO-SMCs (Figure S2A, S2B, S2C). The total amount of MAPK protein remained unchanged with estrogen stimulation. To determine whether MAPK are involved in estrogen-altered cell viability in WT-SMCs and CSE-KO-SMCs, U0126 (a selective inhibitor of the MEK/ERK signaling pathway), SB203580 (a p38 MAPK inhibitor), SP600125 (a JNK inhibitor), and ICI 182780 were used in the following experiments. We first validated that treatment of WT-SMCs and CSE-KO-SMCs with MAPK inhibitors significantly suppressed estrogen-induced phosphorylation of ERK, p38 MAPK and JNK (Figure S3A, S3B, S3C). Blockage of ER activation by ICI 182780 significantly inhibited estrogen-induced phosphorylation of MAPK (Figure S3A, S3B, S3C). SB203580 and SP600125 did not affect estrogen-changed cell viability, but U0126 decreased the viability of both WT-SMCs and CSE-KO-SMCs (Figure 6A and 6B). We further found that inhibition of ERK phosphorylation did not affect estrogen-altered expression of cyclin D1 in both WT-SMCs and CSE-KO-SMCs (Figure S4A and S4B). Therefore, MAPK may not be involved in the effects of estrogen on the growth of both WT-SMCs and CSE-KO-SMCs.

## Discussion

The incidence of proliferative cardiovascular diseases is higher among men than women, but it rises in aging women after the menopause, suggesting a protective role of estrogen [2], [21]. H_2_S is a novel gasotransmitter and provides cardiovascular protection [7], [8], [12], [15], [16], [22], [23]. Both H_2_S and estrogen can inhibit cell proliferation [3], [4], [7], [8], [13]. However, the interaction between estrogen and H_2_S as well as its effects on cardiovascular function are not clear.

Estrogen has been used in previous studies at concentrations from 1 pM to 1000 nM [1], [24], [25]. Our present study uses estrogen at the similar range and in most of our experiments 100 nM estrogen was used. In this study, we demonstrated that estrogen significantly reduced the growth of WT-SMCs which produce H_2_S normally, but increased that of CSE-KO-SMCs which lack the endogenous H_2_S production (Figure 1). High dose of estrogen (1000 nM) inhibited both cell proliferations, suggesting very higher concentration of estrogen displays a toxic effect and kills the cells via a nonspecific pathway. It is also well known that estrogen at 1000 nM is far beyond of pathophysiological range. We previously showed that over-produced H_2_S derived from CSE overexpression reduced cell growth and CSE deficiency led to a significant increase in the growth of cultured SMCs [7], [8], [26]. Estrogen may inhibit cell growth by stimulating CSE/H_2_S system. However, we found that estrogen has little effect on CSE expression and H_2_S production. Therefore, it is endogenous H_2_S level that decides the pro- or anti-growth effect of estrogen on SMCs. However, the mechanism by which H_2_S assumes this switch role is not clear.

It is generally accepted that estrogen receptors (ERs) mediate the anti-growth effect of estrogen on SMCs [9], [27]. Two types of estrogen receptor exist: ERs and estrogen G protein-coupled receptor (GPER) [27], [28]. ERs include ERα and ERβ, which are the members of the nuclear hormone family of intracellular receptors [27]. GPER belong to G protein-coupled receptor [28]. GPER is a new discovered ER at the moment and need to explored in the future. In present study, we only observed the change of ERα and ERβ, both of which are DNA-binding transcription factors and expressed in SMCs [27]. Estrogen has been shown to regulate gene transcription by activating both ERα and ERβ [9], [27], [29]. ERβ differs from ERα in 2 important functional domains [29]. The N-terminus containing the activation function-1 (AF-1) domain of ERα and ERβ has only 30% homology, and the hormone-binding (HBD) domains of ERα and ERβ have only 53% homology [29]. Here, we demonstrated that the expression of ERα was significantly reduced in the absence of CSE expression but induced by exogenously applied H_2_S. However, CSE deficiency had no effect on the expression of ERβ, suggesting that H_2_S may regulate ERα expression by targeting at the AF-1 or HBD domain. It is not clear at this moment how H_2_S regulates ERα expression. H_2_S can post-translationally modify the proteins by *S*-sulfhydration [30], [31], and it is possible that H_2_S may *S*-sulfhydrate some transcript factors or proteins, which subsequently stimulate ERα expression.

Altered ERα expression or activity contributes to different regulatory roles of estrogen in cell growth between WT-SMCs and CSE-KO-SMCs. First, blockage of ERs activity by ICI 182780 reversed the anti-growth effect of estrogen on WT-SMCs (Figure 3A). Second, supplement of WT-SMCs with exogenously applied H_2_S at 100 µM aggravated the anti-growth effect of estrogen (Figure 2D). Third, when ERα expression was knockdown by ERα-specific siRNA in WT-SMCs, estrogen had no effect on SMC growth (Figure 3, B and D). Fourth, overexpression of ERα overturned the pro-growth effect of estrogen in CSE-KO-SMCs (Figure 3E). These discoveries indicate the interaction of H_2_S and ERα plays a key role in the effects of estrogen on SMC growth.

ERs belong to ligand-activated transcription factors [27], [29]. Cyclin D1 is a downstream gene of ERs [9]. The present study found that estrogen at 100 nM significantly inhibited cyclin D1 expression in WT-SMCs, but increased cyclin D1 expression in CSE-KO-SMCs (Figure 4, A and B). Cell cycle progression is determined by the formation of protein complexes among cyclins and cyclin-dependent kinases (cdks), and the association of cyclin D1 with cdk2, cdk4, and cdk6 determines early G1 progression [8]. We have shown before that cyclin D1 is inhibited by exogenously applied H_2_S or CSE overexpression but induced by CSE deficiency, all of which contribute to the regulatory role of H_2_S on SMC growth [8], [13]. Here, we further found that cyclin D1 mediated the anti-growth effect in WT-SMCs and the pro-growth effect in CSE-KO-SMCs by estrogen treatments (Figure 4 and 5), pointing to the critical role of endogenous H_2_S in ERα-mediated cyclin D1 expression following altered cell growth.

In summary, our study for the first time demonstrates that H_2_S mediates estrogen-inhibited proliferation of SMCs via selective activation of ERα/cyclin D1 pathways. The elucidation of the interaction between H_2_S and estrogen on the regulation of SMC growth would provide new insight for prevention and treatment of proliferative cardiovascular disease such as atherosclerosis. The physiological and pathological implications of the interactions between H_2_S and estrogen in proliferative vascular diseases merit further investigation.

## Supporting Information

Figure S1 **Estrogen had no effect on CSE expression and H_2_S production.** ***A***, The expression of CSE was not changed by estrogen treatments. After WT-SMCs were incubated with the indicated concentration of estrogen for 72 h, the cells were collected and subjected to western blotting with anti-CSE antibody. ***B***, Estrogen did not affect H_2_S production. After WT-SMCs were treated with the indicated concentration of estrogen for 72 h, H_2_S production rate was measured. All the results were representative of five individual experiments.
(DOC)

Figure S2 **Different activation of MAPK by estrogen between WT-SMCs and CSE-KO-SMCs.** Time course of the activation of ERK (***A***), p38 (***B***), and JNK (***C***) induced by estrogen. The graphs represent the optical density of the bands of phospho-MAPK normalized with the expression of total-MAPK. All the experiments were repeated for three times. * p<0.05 *vs*. 0 min of WT-SMCs; ^#^ p<0.05 *vs*. 0 min of CSE-KO-SMCs; ^$^ p<0.05 *vs*. WT-SMCs same time group.
(DOC)

Figure S3 **Mediation of ERα in estrogen-induced activation of MAPK.** Cells were treated with 10 µM ICI 182780 or 10 µM U0126 (a MEK/ERK inhibitor) (***A***) or 10 µM SB 203580 (a p38 MAPK inhibitor) (***B***) or 10 µM SP 600125 (a JNK inhibitor) (***C***) for 30 min and followed by 100 nM estrogen treatments for another 30 min. The data were from three independent experiments. * p<0.05 *vs*. control group; ^#^ p<0.05 *vs*. estrogen group; ^$^ p<0.05 *vs*. WT-SMCs same group.
(DOC)

Figure S4 **Inhibition of ERK had no effect on estrogen-altered cyclin D1 expression.** WT-SMCs (***A***) and CSE-KO-SMCs (***B***) were pretreated with 10 µM U0126 for 30 min followed by 100 nM estrogen for 72 h. The data were from three independent experiments. * p<0.05 *vs*. control group.
(DOC)
