# Supplementary figures and images for: Interaction of Hydrogen Sulfide and Estrogen on the Proliferation of Vascular Smooth Muscle Cells
Source: PLoS One. 2012 Aug 3;7(8):e41614. doi: 10.1371/journal.pone.0041614 (PMC3411693; doi:10.1371/journal.pone.0041614)

**
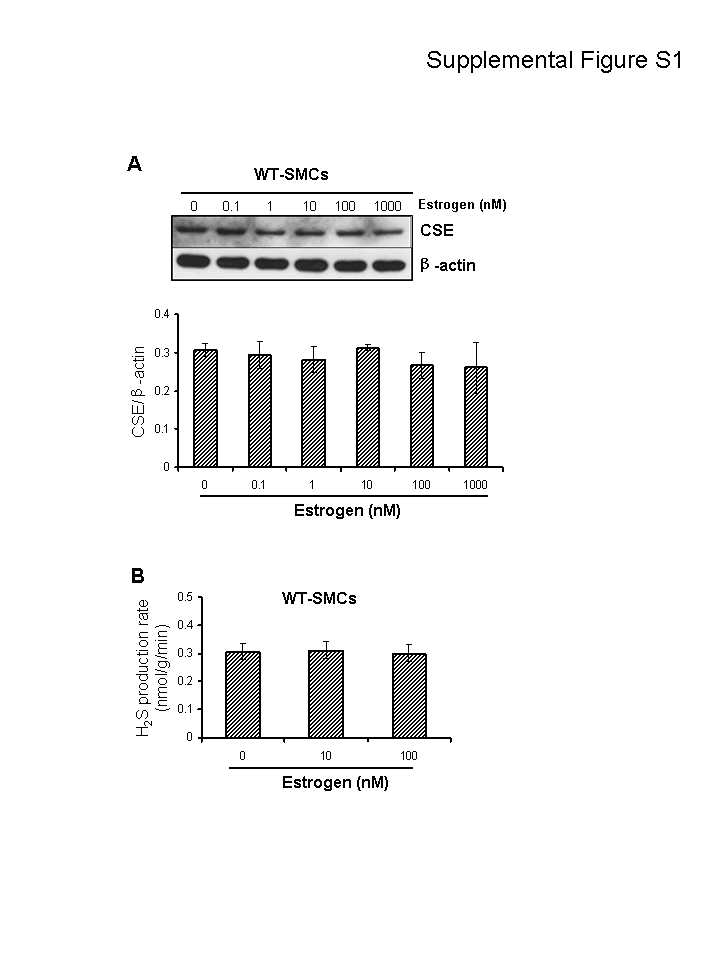
**

Supplement: Figure S1 — Estrogen had no effect on CSE expression and H2S production. A, The expression of CSE was not changed by estrogen treatments. After WT-SMCs were incubated with the indicated concentration of estrogen for 72 h, the cells were collected and subjected to western blotting with anti-CSE antibody. B, Estrogen did not affect H2S production. After WT-SMCs were treated with the indicated concentration of estrogen for 72 h, H2S production rate was measured. All the results were representative of five individual experiments. (DOC) [file pone.0041614.s001.doc]

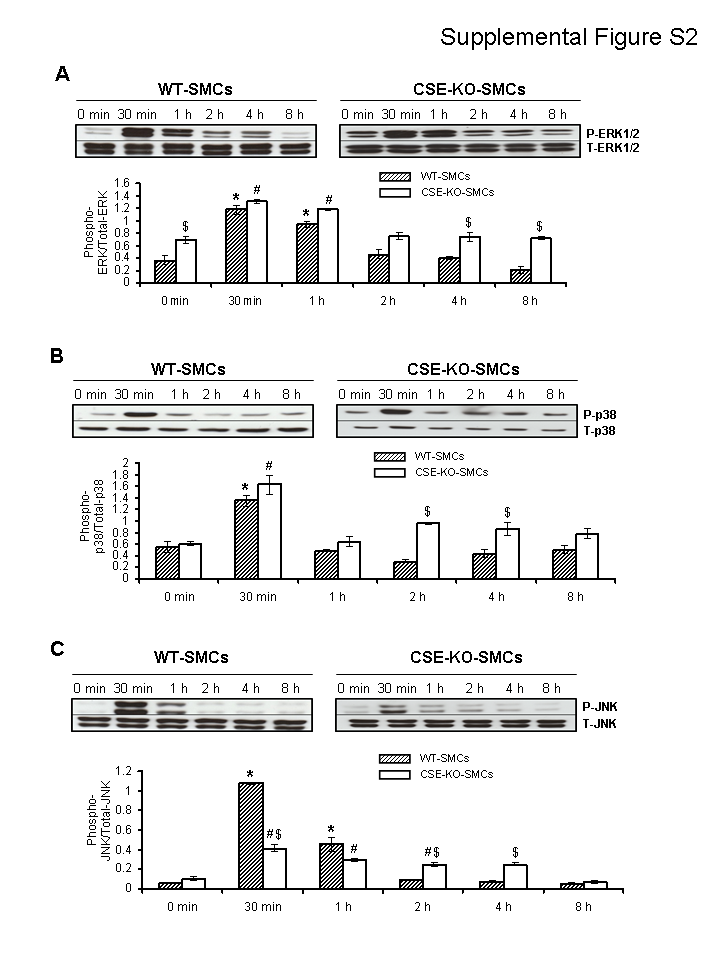

Supplement: Figure S2 — Different activation of MAPK by estrogen between WT-SMCs and CSE-KO-SMCs. Time course of the activation of ERK (A), p38 (B), and JNK (C) induced by estrogen. The graphs represent the optical density of the bands of phospho-MAPK normalized with the expression of total-MAPK. All the experiments were repeated for three times. * p<0.05 vs. 0 min of WT-SMCs; # p<0.05 vs. 0 min of CSE-KO-SMCs; $ p<0.05 vs. WT-SMCs same time group. (DOC) [file pone.0041614.s002.doc]

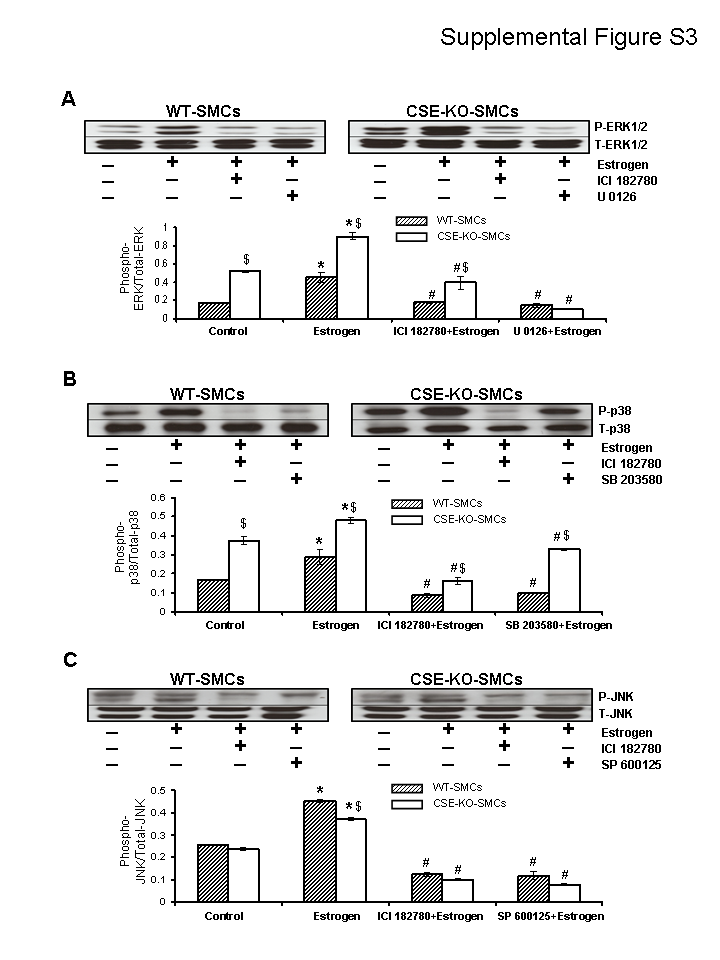

Supplement: Figure S3 — Mediation of ERα in estrogen-induced activation of MAPK. Cells were treated with 10 µM ICI 182780 or 10 µM U0126 (a MEK/ERK inhibitor) (A) or 10 µM SB 203580 (a p38 MAPK inhibitor) (B) or 10 µM SP 600125 (a JNK inhibitor) (C) for 30 min and followed by 100 nM estrogen treatments for another 30 min. The data were from three independent experiments. * p<0.05 vs. control group; # p<0.05 vs. estrogen group; $ p<0.05 vs. WT-SMCs same group. (DOC) [file pone.0041614.s003.doc]

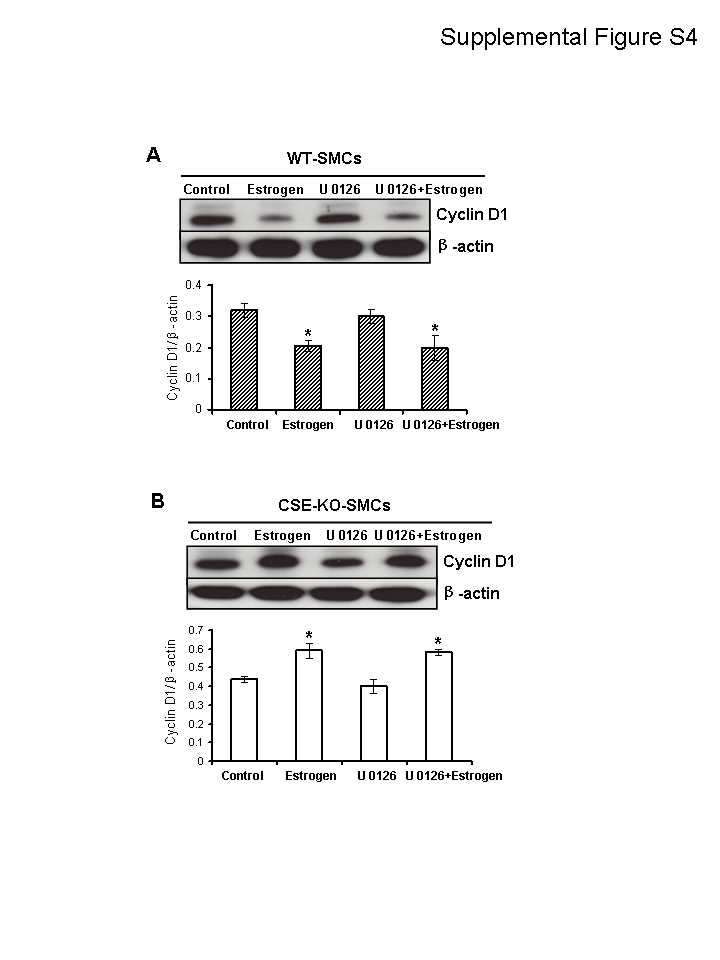

Supplement: Figure S4 — Inhibition of ERK had no effect on estrogen-altered cyclin D1 expression. WT-SMCs (A) and CSE-KO-SMCs (B) were pretreated with 10 µM U0126 for 30 min followed by 100 nM estrogen for 72 h. The data were from three independent experiments. * p<0.05 vs. control group. (DOC) [file pone.0041614.s004.doc]
